# Asymmetric HIV-1 co-receptor use and replication in CD4^+^ T lymphocytes

**DOI:** 10.1186/1479-5876-9-S1-S8

**Published:** 2011-01-27

**Authors:** Samanta A Mariani, Elisa Vicenzi, Guido Poli

**Affiliations:** 1 AIDS Immunopathogenesis Unit, Division of Immunology, Transplantation and Infectious Diseases, San Raffaele Scientific Institute, Milano, Italy; 2 Viral Pathogens and Biosafety Unit, Division of Immunology, Transplantation and Infectious Diseases, San Raffaele Scientific Institute, Milano, Italy; 3 Vita-Salute San Raffaele University, School of Medicine, Milano, Italy

## Abstract

Susceptibility to infection by the human immunodeficiency virus type-1 (HIV-1), both *in vitro* and *in vivo,* requires the interaction between its envelope (Env) glycoprotein gp120 Env and the primary receptor (R), CD4, and Co-R, either CCR5 or CXCR4, members of the chemokine receptor family. CCR5-dependent (R5) viruses are responsible for both inter-individual transmission and for sustaining the viral pandemics, while CXCR4-using viruses, usually dualtropic R5X4, emerge in ca. 50% of individuals only in the late, immunologically suppressed stage of disease. The hypothesis that such a major biological asymmetry is explained exclusively by the availability of cells expressing CCR5 or CXCR4 is challenged by several evidences. In this regard, binding of the HIV-1 gp120 Env to the entry R complex, i.e. CD4 and a chemokine R, leads to two major events: virion-cell membrane fusion and a cascade of cell signaling. While the fusion/entry process has been well defined, the role of R/Co-R signaling in the HIV-1 life cycle has been less characterized. Indeed, depending on the cellular model studied, the capacity of HIV-1 to trigger a flow of events favoring either its own latency or replication remains a debated issue. In this article, we will review the major findings related to the role of HIV R/Co-R signaling in the steps following viral entry and leading to viral spreading in CD4^+^ T lymphocytes.

## Introduction

Infection with the human immunodeficiency virus 1 type-1 (HIV-1) causes a severe and selective depletion of the CD4^+^ T lymphocytes both *in vivo* and *in vitro*. Such a selective tropism and pathogenicity of the virus immediately suggested an important involvement of the CD4 molecule in the infectious process. In 1984, CD4 was described as main receptor (R) for HIV-1 entry into this subset of T lymphocytes [1-3]. Later on, CD4 was also demonstrated to be the main entry R for the virus in mononuclear phagocytes and dendritic cells (DC), although not in astrocytes of the central nervous system [4]. However, the requirement for one or more essential cofactor(s) or Co-R in addition to CD4 for allowing HIV-1 (and HIV-2) entry in target cells emerged rapidly by studies in which non-human cells transfected with human CD4, although capable of binding HIV-1 virions, were not permissive to HIV infection and replication [5-7].

After a decade of research and “false alarms” two 7-transmembrane domain G protein-coupled R belonging to the chemokine R family, i.e. CXCR4 and CCR5, were sequentially identified as the missing cell surface factors conferring competence for entry and replication of the virus into CD4^+^ cells [5,8-11]. Viruses were then classified as R5, X4 or “dualtropic” R5X4 depending on which Co-R they used to infect non-human cells transfected with human CD4 and a panel of human chemokine R, including CCR2 and CCR3, rarely used *in vivo *[12]. R5 viruses were soon demonstrated to be responsible for both inter-individual transmission, regardless of the route of transmission (i.e., sexual, systemic, mother-to-child), and for sustaining the pandemics, whereas CXCR4-using viruses, usually dualtropic R5X4, emerge in ca. 50% of individuals in the late, immunologically suppressed stage of disease. Fuerthermore, such a “switch” in Co-R usage (that should be more correctly referred to as an extension in most cases) almost exclusively occurs in individuals infected with subtype B viruses, dominant in North America, Europe and Australia [13]. However, a superior cytopathicity of CXCR4-using viruses is well demonstrated and results in an accelerated progression to AIDS and death if the infection is not halted by anti-retroviral therapy (ART) [13,14].

These epidemiological observations pose the question of whether such a disproportionate asymmetry in HIV-1 Co-R use is exclusively determined at the virion entry level by availability of CCR5 vs. CXCR4 expression on the surface of CD4^+^ cells or whether R/Co-R mediated signaling also influences the capacity of the virus to efficiently replicate in its own target cells. In this regard, the viral entry step in the retroviral life cycle was demonstrated not to be dependent upon G-coupled R signaling since mutations in CCR5 ablating its signal transduction capacity did not affect its Co-R activity in transfected cells [15,16]. However, triggering of chemokine signaling could potentially contribute to viral replication at a post-entry level, for example by promoting or repressing proviral transcription. This article will briefly overview what has been learned on this second potential function of CCR5 vs. CXCR4 in CD4^+^ T lymphocytes.

## Co-R expression and state of activation in peripheral blood and lymphoid tissue associated CD4^+^ T lymphocytes

From the bone marrow, T lymphocyte precursors reach the thymus where they become terminally differentiated into CD4^+^ and CD8^+^ T cells and are then exported into the bloodstream [17]. Concerning the expression of HIV-1 Co-R in thymocytes, CXCR4 is abundantly expressed by both thymocytes and epithelial cells while CCR5 is detected only on a minority of mature thymocytes, as reviewed in [18]. Yet, although X4 infection and cytopathicity of thymocytes clearly occurs, R5 viruses can infect and replicate efficiently in this organ [18].

Phenotypically, peripheral blood naïve T cells are characterized by the surface expression of three key molecules: the RA isoform of the CD45 antigen, the L-selectin ligand (CD62L) and CCR7, a chemokine R [19,20]. Immature DC, like Langherans’cells in the skin, express CCR7 and can therefore migrate, like T cells, to the lymph nodes in response to CCR7L, CCL19 and CCL21, expressed by lymph nodes associated cells. Memory T cells can be divided into central memory (T_CM_) and effector memory (T_EM_) cells distinct for phenotypic and functional properties [21]. In particular, human T_CM_ are CD45RO^+^ and constitutively express CCR7 and CD62L that are required for cell extravasation through high endothelial venules and migration to T cell areas of secondary lymphoid organs [19,20]. In contrast, human T_EM_ have lost the constitutive expression of CCR7, are heterogeneous for CD62L expression, and display characteristic sets of chemokine R and adhesion molecules required for homing to inflamed tissues [21]. The levels or cell surface density of both CD4 and HIV Co-R in these different subsets of T cells may play a significant role in the efficiency of virion-cell membrane fusion and, consequently, their susceptibility to infection [22].

The importance of the density of CD4 and Co-R for HIV infection was investigated also in cell types different than T cells. In HeLa-CD4/CCR5 cells stably expressing minimal concentrations of CCR5 on the cell surface R5 HIV-1 infection occurred more efficiently in concomitance of “high” levels of CD4 expression, suggesting that a minimal number of CCR5 molecules are required when cell surface levels of CD4 reach a sufficient density [22]. At lower CD4 concentrations, higher numbers of CCR5 molecules were indeed necessary to achieve the same high efficiency of viral replication [22]. In this regard, the levels of CD4 and Co-R expression in peripheral blood T cells can vary from 2- to 5-folds among different donors and also depend on the activation status of the cells. CXCR4 is usually expressed by a higher fraction of CD4^+^ T lymphocytes than CCR5; conversely, the levels of CCR5 expression on the cells that stain positive for this chemokine R are usually 2-3 fold greater than those of CXCR4 [23]. In addition, HIV-1 has been shown to deplete preferentially HIV-specific CD4^+^ T cells, likely via a “double kiss” through the CD4/Co-R complex facilitated by the virological synapse established by T cell receptor (TCR)/MHC class II interaction with viral peptides expressed by the infected cells [24]. Thus, it is difficult to decipher the dynamics of HIV-1 in infected individuals simply on the basis of CCR5 and CXCR4 expression on CD4^+^ T cells.

CCR5 is expressed preferentially by the CD45RO^+^ subset of T_EM_, whereas CXCR4 is more dense on the surface of naïve CD45RA^+^ T cells [25]. Activated/memory T cells can traffic to peripheral lymphoid organs at mucosal surfaces and form conjugates with DC or macrophages at these sites [26], thus representing early targets for infection and spreading of R5 viruses [27]. Although naïve T cells show lower levels of CCR5 expression and higher levels of CXCR4 in comparison to memory cells [25], they were shown to possess a higher *in vitro* susceptibility to R5 HIV-1 infection than memory T cells following stimulation with immobilized anti-CD3 plus anti-CD28 Ab. This paradoxical observation was explained by the higher capacity of memory T cells to secrete CCR5 ligands, i.e CCL3, CCL4 and CCL5, acting as antagonists of HIV-1 infection, following TCR cell stimulation [28]. In addition, CD4^+^ T cell activation by anti-CD3 plus anti-CD28 immobilized Ab induced a downregulation of CCR5 while enhancing secretion of its ligands [29-31]. Furthermore, sustained CD28 signaling, as well as IL-4 [32,33], can upregulate the expression of CXCR4 and, consequently, favor X4 HIV-1 infection and replication in activated T cells [34]. Conversely, IL-2 stimulation of T cells was shown to induce an increased expression of CCR5 concomitantly with the secretion of CCR5 ligands whereas CD40L expression by T cells resulted in increased secretion of CCR5 ligands by macrophages and DC with selective inhibition of R5, but not of X4 HIV-1 infection [35].

Concerning tissue-associated CD4^+^ T lymphocytes, X4 HIV-1 replication was earlier shown to be more efficient than that of R5 viruses in suspensions of human lymphoid tissue [36]. This observation was confirmed in histological cultures of lympoid tissue blocks (a biological system that maintains, at least in part, the integrity of the lymphoid organs therefore better reflecting the *in vivo* situation than cell suspension of meshed tissue) and was correlated to a higher percentage of CXCR4^+^ cells vs. CCR5^+^ cells as well as to the “constitutive” production of CCR5 ligands [37]. In later studies by the Margolis’ team, both rectosigmoidal and cervico-vaginal tissues were shown to be more prone than tonsillar tissue to R5 infection likely because of the high prevalence of R5 targets and a reduced chemokine production and R blockade [38,39]. Additional evidence that X4 HIV-1 can replicate with higher efficiency than R5 in both cord blood- and adult-derived PBMC was also reported [40]. In contrast, Yamamoto and colleagues suggested that a selective spreading of R5 vs. X4 HIV-1 occurred in the context of DC-T cell co-cultures. The superior efficiency of R5 vs. X4 viruses in DC-T cell spreading was shown to be dependent upon the state of activation of CD4^+^ T cells and not consequent of a higher efficiency by virus to infect DC [41] (**Table **1). In this regard, R5 HIV-1 replicated more efficiently than the X4 viruses in peripheral blood derived primary CD4^+^ T cells expressing *in vivo* levels of CCR5 on their surface [42]. In particular, Fiser and colleagues showed that CCR5 expression did not vary significantly with time in primary CD4^+^ T cells maintained in culture in the absence of stimuli. This was in contrast to CXCR4 density that increased by 10 fold after 24 h of culture likely consequently to the absence of CXCL12-dependent CXCR4 internalization [42]. Indeed, when lymphocytes were co-cultivated with 293T cells transduced with a lentiviral vector expressing CXCL12, the R5 HIV-1 replicated more efficiently than the X4 virus [42].

**Table 1:** Controversial results on the capacity of R5 vs. X4 HIV-1 to replicate in primary CD4+ T lymphocytes in vitro

CELL TYPE	STIMULATORY CONDITIONS	KEY REFERENCES
**X4=R5**		

Lymphoid histo-cultures	none	Grivel et al. (40)

**X4>R5**		

Lymphocyte tissue suspensions	none	Eckstein et al. (38)
Cord blood CD4^+^ T cells	PHA+IL-2	Sundaravaradan et al. (42)
PBMC-derived Th2 cell clones	Anti-CD3 mAb or Der P1+ IL-2	Maggi et al. (51) Annunziato et al. (52)
PBMC-derived Th2 cells	Anti-CD3+anti-CD28 mAb	Gosselin et al. (54)

**R5>X4**		

PBMC-derived CD4^+^ T cells	none	Fiser et al. (44)
PBMC-derived Th1 cell clones	Anti-CD3 mAb or Der P1+ IL-2	Maggi et al. (51) Annunziato et al. (52)
Cord blood derived Th0, Th1, Th2 cells	Anti-CD3 mAb + IL-2	Vicenzi et al. (55)
PBMC-derived Th1 and Th2 cell clones	Anti-CD3 mAb+ IL-2	Vicenzi et al. (55)
PBMC-derived CD4^+^ T cells co-cultured with DC	Anti-CD3+anti-CD28 mAb	Yamamoto et al. (43)
Naïve and memory peripheral T cells	None	Bleul (25)
Recto-signoidal histocultures	None	Grivel et al. (40)
Cervico-vaginal histocultures	Culture medium	Saba et al. (41)

* after normalization per number of CCR5 vs. CXCR4 expressing cells.

Thus, depending on the *in vitro* stimulation of CD4^+^ T cells, the susceptibility to R5 and X4 HIV-1 infection can vary. In particular, R5 viruses appear to possess a superior replicative capacity than X4 HIV in homeostatic conditions (i.e. low cell activation status and *in vivo* levels of the respective chemokine R), whereas a sustained and prolonged *in vitro* T cell activation leading to the overexpression of CXCR4 on cellular surface tilts the balance in favor of X4 viruses in terms of replicative potential.

## Infection and Co-R expression in CD4^+^ T lymphocyte subsets

CD4^+^ T lymphocytes may undergo functionally distinct programs of polarized activation and differentiation, defined as Th1/Th2 (or type 1/type 2) T cell helper pathways reflecting a preferential activation of cell-mediated vs. humoral immunity, respectively [43]. In regard to HIV infection, a shift from Th1 to Th0/Th2 mediated immune response has been early postulated as crucial determinant of both the susceptibility of most individuals to infection and of the progression of disease [44] by analogy with the dichotomous roles played by polarized immune responses in parasite infection [45]. In the mid 90’s a differential expression of chemokine R was associated with Th1 and Th2 cells; in particular, CXCR3, CCR1 and CCR5 were related to Th1 polarization while CXCR4, CCR3 and CCR4 expression was associated with Th2 cells [46].

CCR5 is almost undetectable in resting T cells, although it is promptly upregulated on the surface of Th1 cells by the related cytokines IL-12 and IFN-γ [46-48]. Conversely, CCR5 was shown to be downregulated by the Th2 cytokine IL-4 that, in contrast, upregulated CXCR4 expression on the surface of both Th2 cells and other cell types [32,33]. Annunziato et al. reported that acute infection of Th1 and Th2 cells indeed reflected the predictable pattern of differential HIV-1 Co-R expression, with Th1 and Th2 cells supporting efficiently R5 and X4 virus replication, respectively, although Th1 cells were reported to limit R5 HIV-1 spreading via upregulated secretion of CCR5 binding chemokines [49-51]. However, this study was focused on the early kinetics of HIV-1 replication during the first week of infection. More recently, Th1 cells have been described as expressing CXCR3, CCR5 and CXCR4 but not CCR6 and being relatively resistant to R5 and X4 HIV in vitro [52]. In the same study, Th2 cells were shown to express CCR4, but not CCR6 or CCR5, and were permissive to X4 HIV only [52].

**Figure 1:**
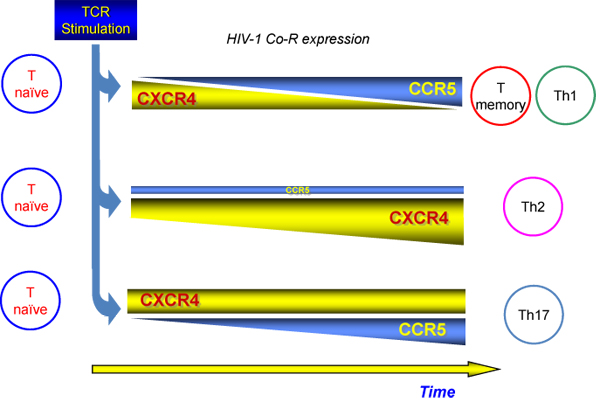
**HIV-1 replication and co-receptor use in naive, memory and Th subsets.** Both Naive and Th2 cells are characterized by high level of CXCR4 expression that is upregulated by IL-4. Memory and Th1 cells show surface levels of CCR5 higher than those of CXCR4, whereas Th17 cells express both Co-R.

**Figure 2:**
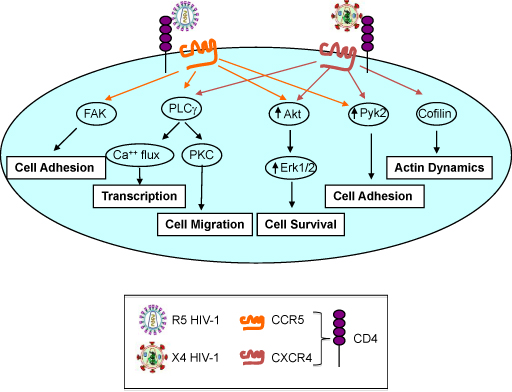
**Differential cell activation pathways triggered by R5 and X4 HIV-1 in CD4^+^ T cell.** Both R5 and X4 HIV-1 gp120 Env engagement of CD4 and Co-R trigger signal transduction events leading to cell activation, host gene transcription, cell migration, survival and adhesion. R5 HIV-1 specifically induces adhesion via activation of FAK whereas X4 viruses modulate actin dynamics via activation of cofilin.

An earlier study from our group looked at both polarized and unpolarized T cells of different origin, including classical Th1, Th2 or Th0 (unpolarized) T cell clones originated from adults peripheral blood or non-immortalized T cell lines derived from cord blood cells (particularly rich in naïve T cells) maintained for 14-20 days after polarization in medium containing IL-2 before exposure to R5 or X4 HIV-1. We have earlier observed that only R5, but not X4, HIV-1 efficiently replicated in both polarized and unpolarized T cell lines or clones in the face of the higher levels of expression of CXCR4 observed particularly in Th2 cells [53]. The lack of X4 HIV replication was not sustained by endogenous expression of CXCL12 and was reproduced with viruses derived from infectious molecular clones (pNLA4-3 and pNL-Ad8) sharing the same backbone except for the *env*-coding region, X4 and R5, respectively [53]. Of interest was the fact that infection by both R5 and X4 HIV-1 occurred with comparable efficiency in these primary cells up to 72 h post-infection when R5 viruses began to spread with an approximately 100-fold higher efficiency than X4 viruses [53]. However, re-stimulation of infected T cells by anti-CD3 mAb showed a dichotomous effect on R5 and X4 infections in that, on the one hand, strongly induced X4 HIV-1 replication, while, on the other hand, did not affect significantly the already efficient capacity of R5 viruses to propagate in these T cells [53]. We interpreted these results as suggestive of a superior capacity of CCR5 in delivering a cell-activating signal required for efficient HIV-1 spreading in T cells that is lacking when the cells are infected with an X4 virus (**Figure **1). This interpretation is supported by earlier evidence showing that R5 gp120 Env trimers can deliver a qualitatively or quantitatively different signal to T cells resulting in Ca^++^-dependent signaling [54-56], as reviewed elsewhere in this issue. Indeed, Ca^++^-dependent signaling was earlier shown in different model systems to activate HIV expression in chronically infected cells [57] (**Figure **2).

Concerning the novel subset of Th17 cells, which play a beneficial role in immunity against bacteria and fungi and contribute to the pathogenesis of autoimmune diseases, it has recently been shown that they are characterized by co-expression of CCR4 and CCR6 [52,58]. In addition, they express both CCR5 and CXCR4 therefore being permissive to R5 and X4 HIV replication, as reviewed in ref [59].

Although CD4^+^ T regulatory cells (Tregs) are believed to play an important role in HIV pathogenesis [60], as illustrated by their involvement in the immune response in the apathogenic model of African Green Monkeys to SIV infection [61] as well as in pathogenic SIV infection of macaques by FoxP3+ CD8+ T cells [62], no specific studies have been designed to address their potential role in R5 vs. CXCR4-dependent HIV-1 infection.

The observation that R5 viruses are endowed with a higher capacity to replicate in CD4^+^ T cells than X4 viruses, together with their ability to replicate in mononuclear phagocytes [63], provides a potential correlation of their superior capacity of establishing a productive infection after viral transmission and during the asymptomatic stage of disease.

## CD4/Chemokine Co-R signaling in HIV infection

The sequential interaction between gp120 Env with CD4 and the chemokine R triggers different cell activation signals. In particular, the interaction of HIV-1 with CCR5 leads to the phosphorylation of the focal adhesion kinase, of CCR5 itself as well as of their association [64], activation of ion channels [56], Ca^++^ mobilization [54], tyrosine (Tyr) phosphorylation of the Tyr kinase Pyk2 [65], downregulation of intracellular cAMP [66], and induction of chemotaxis [55]. CCR5 signaling has also been known to trigger distinctive signaling cascades that activate kinases and transcription factors associated with cell activation [67,68]. For example, R5 gp120 Env can induce the expression of genes either belonging to Mitogen-Activated Protein Kinase (MAPK) pathways and others regulating the cell cycle [68]. R5 gp120 Env can also activate members belonging to the Nuclear Factor of Activated T-Cells (NFAT) family of transcription factors and induce their translocation to the nucleus [69], enhancing viral transcription directly from the LTR promoter. Moreover, the interaction of X4 viruses with CXCR4 leads to the activation of an actin-depolymerization factor, cofilin, that increase the cortical actin dynamics in resting CD4^+^ T cells, leading to HIV replication [70] (**Figure **2).

The direct involvement of chemokine Co-R signaling in HIV-1 infection has been investigated soon after the identification of CXCR4 and CCR5 as obligatory entry Co-R. Cocchi and colleagues reported that inhibition of Co-R signaling through pertussis toxin (PTX) neither inhibited HIV-1 replication nor the ability of chemokines such as CCL5 to block HIV-1 entry into PM1 cells [15]. However, this phenotype was likely due to the unresponsiveness of this cell line to PTX (M. Alfano, personal communication). Both PTX and its oligomeric nontoxic derivative PTX-B were actually shown to be potent inhibitors of R5 HIV-1 entry as well as of both R5 and X4 virus replication in different target cells, including primary T and macrophage cells, secondary lymphoid histocultures and acutely or chronically infected cell lines [71,72].

Three CCR5 mutants of the highly conserved Asp-Arg-Tyr (DRY) motif in the second intracellular loop were required for agonist-induced CCR5 activation, but showed minimal effects on viral entry and replication in Hela-CD4 cells [16]. Similar mutations in CXCR4 also failed to affect its Co-R capacity *in vitro *[73]. Moreover, expression of either WT or signaling-deficient CCR5 molecules by lentivirus-mediated gene transfer into primary T cells and macrophages from *ccr5Δ32* homozygous individuals resulted in comparable efficiency of virus replication [74], supporting the concept that signaling via CCR5 or CXCR4 was not required for viral entry and, accordingly to at least some authors, replication into host cells.

In contrast, several studies have provided evidence for an important role of gp120 Env-mediated signaling in HIV-1 replication. By avoiding mitogen-dependent pre-activation of T cells, G-protein signaling triggered during R5 infection significantly facilitated virus replication in primary CD4^+^ T cells [75]. Similarly, IL-2 stimulation of PBMC in the absence of mitogen pre-stimulation resulted in the efficient replication of “macrophage-tropic” (essentially, R5) HIV-1 strains in a cytokine- and monocyte-dependent manner [76]. Moreover, stimulation with viral gp120 trimers triggered virus replication in cultures of resting CD4^+^ T cells of infected individuals [77]. In particular, a direct correlation was observed between the capacity of gp120 Env to initiate signaling and the ability of the virus to replicate in the cells [54]. Furthermore, a signaling-deficient R5 HIV-1 entered monocyte-derived macrophages (MDM) but failed to replicate because of a block occurring at a post-entry level [54]. Before this study, Popik and colleagues, using a monomeric gp120 Env, demonstrated that binding of HIV-1 to CD4 in T cells activates the MAPK/extracellular signal-regulated kinase (MEK) pathway, stimulates the activation of transcription factors (i.e., AP-1, NF-κB and C/EBP) with consequent expression of inflammatory genes [78]. This study also showed that triggering of this signaling pathway was independent of HIV-1 binding to the chemokine R in that could be induced in CD4^+^ cells by both X4 and R5 viruses [78].

A new family of chemokine inhibitors capable of blocking chemokine R signaling without affecting R binding, termed Broad Spectrum Chemokine Inhibitors (BSCI), was recently described [79]. BSCI blocked HIV-1 replication, without inhibiting gp120 Env interaction with CCR5 or CXCR4 in THP-1 monocytic and Jurkat T lymphocytic cell lines, respectively [79]. These findings together suggest that although chemokine Co-R signaling is not required for the efficient entry of R5 and X4 viruses into target cells, it likely plays an important role in order to guarantee efficient virus replication by affecting one or more post-entry steps in the virus life cycle. Indeed, CCR5 ligation might trigger a series of events that could influence cell survival or cell death. In this regard, engagement of CCR5 human monocytes by either HIV-1 or its ligands prior to cellular exposure to apoptotic agents such as the CdCl_2_ significantly reduces the apoptosis [80]. However, no studies has yet determined whether CXCR4 signaling can also confer resistance to apoptosis in monocyte/macrophages independently of immune activation.

## Conclusion

Although a definitive model explaining the differential capacity of R5 and X4 viruses to replicate in CD4^+^ lymphocytes cannot be still drawn, it is important that the search for correlates of such a relevant difference between these two amazingly similar pathogens with so different outcomes for human health (if HIV-1 could only rely on CXCR4 the infection and disease would likely be much more contained and preventable) will still continue.

## Competing interests

No conflicts of interests exist for all the authors and the matter discussed in the article.
